# Mid-term safety and effectiveness of macular peeling one month after intravitreal dexamethasone implant for tractional diabetic macular edema

**DOI:** 10.1038/s41598-023-32780-5

**Published:** 2023-04-12

**Authors:** Francesco Pignatelli, Alfredo Niro, Matteo Fallico, Fedele Passidomo, Samuele Gigliola, Annalisa Nacucchi, Vincenza Bonfiglio, Michele Reibaldi, Giuseppe Addabbo, Teresio Avitabile

**Affiliations:** 1 Eye Clinic, Hospital “SS. Annunziata”, ASL TA, Via F. Bruno, 1, 74010 Taranto, Italy; 2 Department of Ophthalmology, University of Catania, Catania, Italy; 3 Department of Experimental Biomedicine and Clinical Neuroscience, University of Palermo, 90127 Palermo, Italy; 4 Department of Surgical Sciences, Eye Clinic Section, University of Turin, Turin, Italy

**Keywords:** Retinal diseases, Drug discovery, Drug delivery

## Abstract

Macular peeling combined or followed by intravitreal dexamethasone implant (DEX-i) was recommended as an efficacy approach for tractional diabetic macular edema (tDME). Knowing the synergistic effect of cataract surgery and DEX-i one month earlier in eyes with DME, we compared Epiretinal Membrane/Inner Limiting Membrane (ERM/ILM) peeling preceded by DEX-i one month before versus ERM/ILM peeling alone for the treatment of tDME. A retrospective study on patients affected by tDME who underwent ERM/ILM peeling one month after DEX-i (n = 11; Group A) or ERM/ILM peeling alone (n = 10; Group B) was performed. Longitudinal comparison of best-correct visual acuity (BCVA), central retinal thickness (CRT), and intraocular pressure (IOP) between the time of surgery (T_0_) and each time point (months 1,3,5,6) within and among the groups were assessed. To evaluate the repeated measurements of BCVA, CRT, and IOP, a linear mixed-effects model was used. In Group A, DEX-i significantly improved mean BCVA and CRT (*P* < 0.001) just after 1 month (T_0_). After ERM/ILM peeling, mean BCVA and CRT significantly improved from month 1 in Group A and month 3 in Group B. Mixed model revealed a significant difference in BCVA (P ≤ 0.0001) and CRT (P ≤ 0.02) at different time-points among the groups with better results in Group A. Neither complications nor uncontrolled IOP increase was detected. ERM/ILM peeling confirmed its effectiveness in treating tDME. DEX-i performed one month before surgery seemed to be a safe approach and ensured a greater and faster recovery considering functional and tomographic parameters.

## Introduction

Diabetic macular edema (DME) affects approximately 6.8% of the diabetic population^1^ and 20% of the patients with diabetic retinopathy^2^, being the major cause of vision loss in these patients^3,4^^.^

The incidence of epiretinal membrane (ERM) in DME patients has been reported to be 13–34% on structural optical coherence tomography (OCT) imaging^5–8^ and 46.9% on En-face OCT imaging^9^.

Vitrectomy may be considered when there is little or no response to non-surgical treatments^10,11^ or in cases in which DME is associated with vitreomacular traction (VMT) or ERM^11–14^. After surgery, the anatomical outcome has been shown to be better than functional recovery^15^. Evidence from the Diabetic Retinopathy Clinical Research Network (DRCR.net) has demonstrated a reduction of the central retinal thickness (CRT) after vitrectomy and ERM peeling in DME eyes with vitreomacular interface abnormality^16,17^. However, the efficacy of improving visual acuity was limited^16,17^ and influenced by predictive factors, including poorer glycemic control^18^, preoperative visual acuity^16^, greater retinal thickness^18^, presence of subretinal fluid^19^, and lack of integrity of outer retinal layers^20,21^.

Combining vitrectomy and nonsurgical treatments, including intravitreal triamcinolone and laser photocoagulation, revealed favorable outcomes, but with short-term efficacy and only in non-tractional DME^22–24^.

The intravitreal dexamethasone implant (DEX-i) (Ozurdex®, Allergan Inc, Irvine, CA, USA) provides a sustained release of corticosteroid for up to 6 months thanks to its biodegradable polymer matrix. DEX-i has been shown to have similar pharmacokinetic profiles and safety in both vitrectomized and nonvitrectomized eyes^25^. Additionally, DEX-i has been associated with different advantages over other sustained-release steroid implants using fluocinolone acetonide or triamcinolone acetonide as a smaller incidence of increase in intraocular pressure (IOP)^26^ and a complete dissolution within the vitreous cavity that avoid surgical removal of the implant.

Recently, Figueira et al. recommended macular peeling plus DEX-i as first-line therapy for DME when traction is present^27^. Furthermore, ERM and Inner Limiting Membrane (ILM) peeling performed in combination with intravitreal DEX-i showed longer-lasting treatment effects, especially in tractional DME (tDME)^28,29^. However, if vitrectomy and simultaneous DEX-i seem to work synergistically, the efficacy of the use of DEX-I one month before ocular surgery, benefiting from its peak of action, was recently observed in patients with DME undergoing cataract surgery^30,31^.

So, the current study aimed to compare functional and anatomical results of macular peeling alone versus peeling preceded by DEX-i one month before surgery in patients with tDME.

## Methods

### Study design and objectives

We conducted a retrospective, comparative, single-center cohort study on 21 patients affected by tDME. Between September 2015 and February 2016, at the Eye Clinic of “SS. Annunziata” Hospital in Taranto, Italy, 11 consecutive patients were treated with DEX-i performed 30 ± 5 days before primary vitrectomy combined with ERM and inner limiting membrane (ILM) peeling (Group A) and compared with 10 consecutive patients treated with primary vitrectomy associated with ERM and ILM peeling alone (Group B). Inclusion criteria were tDME defined as central-involved diabetic macular edema (cystoid pattern, sponge-like pattern, or retinal detachment pattern) associated with ERM, non-proliferative diabetic retinopathy, proliferative diabetic retinopathy previously treated with laser photocoagulation or intravitreal anti-Vascular Endothelial Growth Factor (VEGF) injection.

Exclusion criteria included HbA1c > 9% (75 mmol/mol), untreated proliferative diabetic retinopathy; a history of ocular hypertension or glaucoma; previous or concomitant retinal diseases including retinal vein occlusion, age-related macular degeneration and other conditions that could worsen DME; treatment of DME with intravitreal anti-VEGF in the 3 months before surgery; treatment of DME with intravitreal corticosteroid in the 6 months before surgery, apart from DEX-i implant one month before vitrectomy in Group A; cataract surgery within the past 6 months; prior history of vitreoretinal surgery; uncompleted follow-up.

This retrospective chart review study involving human participants was in accordance with the ethical standards of the institutional and national research committee and with the 1964 Helsinki Declaration and its later amendments or comparable ethical standards. The Human Investigation Committee (IRB) of the Eye Clinic of “SS. Annunziata” Hospital in Taranto, Italy, approved this study.

The objective of the study was to compare the effect of ERM and ILM peeling performed 1 month after DEX-i with ERM and ILM peeling alone in terms of variation of best corrected visual acuity (BCVA) and CRT.

### Surgical procedure

DEX implant 0.7 mg was injected into the vitreous cavity using standard protocol^32^. Twenty-five Gauge (25G) three ports vitrectomy was performed using the Constellation® Vision System (Alcon Laboratories, Fort Worth, TX). Povidone-iodine 5% preparation (Oftasteril, Alfa Intes Industria Terapeutica Splendore S.r.l., Naples, Italy) was applied to the cornea, conjunctival sac, and periocular skin for 3 min before surgery. Peribulbar anesthesia was performed on all patients. Congiuntival displacement with forceps and three 30° oblique incisions 3.5–4 mm from the limbus were performed to insert three valved cannula trocar systems. For posterior visualization, Oculus BIOM 4 (Oculus Surigcal Inc, FL, USA) and a plano-concave contact lens were used. The central core and peripherical vitrectomy were performed in all cases with 5000 cuts per min (cpm) cut-rate and linear aspiration of 0–650 mmHg.

In Group A, the DEX-i was visible floating in the vitreous chamber during vitrectomy, so a lower aspiration rate and slower maneuver were performed to avoid accidentally cutting the implant with the vitrectome. The use of a triamcinolone-based vitreous stainer was avoided in all surgery. ERM and ILM peeling was performed in all patients, using 25-G internal limiting membrane forceps (Alcon Laboratories) after staining with Trypan Blu (TB) 0.15% + Brilliant Blue G (BBG) 0.05% + Lutein 2%solution (DOUBLE DYNE; Alfa Intes Industria Terapeutica Splendore S.r.l., Naples, Italy). A sclerotomy site suture was performed only when needed due to leakage of the wound.

### Assessments

**Figure 1:**
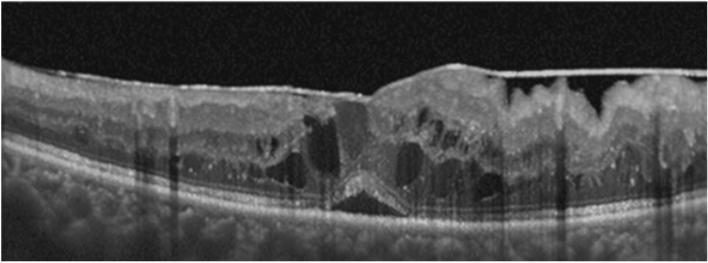
Optical coherence tomography scan showing tractional diabetic macular edema (tDME) with cystoid and retinal detachment pattern, and epiretinal membrane. A representative case.

All participants underwent a complete ophthalmic examination at baseline (before intravitreal dexamethasone implant insertion in Group A; before surgery in Group B), including demographic and anamnestic data and HbA1c levels. At the time of surgery (T_0_) and during the follow-up visits scheduled at months 1,3,5 and 6, BCVA, IOP, and CRT were measured. IOP and BCVA were measured with a Goldmann tonometer and a standardized Early Treatment Diabetic Retinopathy Study protocol, respectively; Early Treatment Diabetic Retinopathy Study Values were converted to the logarithm of the minimum angle of resolution for statistical analysis; CRT was assessed with spectral-domain optical coherence tomography (SD-OCT; CIRRUS, Carl Zeiss, Jena, Germany). CRT was defined as the average thickness of the macula in the central 1-mm Early Treatment Diabetic Retinopathy Study grid. Central involved macular edema was defined as a CRT > 300 microns (Fig. 1).

IOP measurement and all intraoperative and postoperative adverse events were recorded for safety evaluation. Additional non-surgical treatments for postoperative recurrence of DME were identified.

### Statistical analysis

To describe patients’ characteristics at baseline, mean ± standard deviation (SD) was used for continuous variables and counts with percentages for categorical variables. Demographic and baseline characteristics of the two samples were compared using Fisher’s Exact test for categorical variables and Mann–Whitney U Test for quantitative ones. In Group A, the effect of DEX-i on outcomes after 1 month was analyzed using the Wilcoxon Test.

A linear mixed model was used to evaluate repeated measurements of BCVA, CRT, and IOP at each time point within each group and among the groups, and the trajectories of BCVA, CRT, and IOP. A *P* value < 0.05 was considered statistically significant. No formal sample size calculation was performed. All statistical analyses were performed using the software package SAS version 9.1 or higher.

### Ethical approval

All procedures performed in studies involving human participants were in accordance with the ethical standards of the institutional review board as well as the 1964 Helsinki Declaration and its later amendments or comparable ethical standards. The Institutional Review Board (Eye Clinic of “SS. Annunziata” Hospital, Taranto, Italy) approved the study.

### Informed consent

Informed consent was obtained from all individual participants included in the study.

## Results

### Demographic and data before treatment

Patients’ characteristics are reported in Table 1. The study enrolled 21 patients; 11 were assigned to Group A and 10 to Group B. There were 12 female patients in both groups. The mean age was 71,9 ± 5,1 years in Group A and 73,2 ± 8,1 in Group B; in each group, 1 patient had type 1diabetes. Time from diagnosis of diabetes was similar for the two groups, 16 years and 17,8 years, respectively. No difference in HbA1c was observed between the groups. All patients were pseudophakic. There were no significant differences between the two groups regarding mean BCVA, CRT, and IOP when patients were at baseline.

**Table 1:** Demograpic and baseline characteristics.

	Group A (n = 11)	Group B (n = 10)	*p*
Age, years	71,9 ± 5,1	73,2 ± 8,1	0.7
Sex			
Male	5 (45.4)	4 (40)	
Female	6 (54.6)	6 (60)	1*
Type 2 DM	10 (91)	9 (90)	
Type 1 DM	1 (9)	1 (10)	1*
Glycated hemoglobin (%)	7 ± 0,5	7 ± 0,8	0.9
Years from diagnosis of diabetes	16 ± 5,3	17,8 ± 6,5	0.3
BVCA, logMAR	0,82 ± 0,4	0,76 ± 0,2	0.8
CRT, microns	419,1 ± 81,5	408,5 ± 55,4	0.8
IOP, mmHg	16,3 ± 1,7	15,6 ± 1,7	0.5

*DM* Diabetes mellitus, *BCVA* Best corrected visual acuity, *CRT* Central retinal thickness, *IOP* Intraocular pressure. Unless otherwise indicated, values are mean ± SD or no. (%). *p*, Mann–whitney U test, *, Fisher’s exact test.

### Follow-up

The comparisons between the mean of BCVA, CRT, and IOP at each follow-up visit (months 1, 3, 5, and 6) to baseline (T_0_) for each group of treatment were reported.

### Best-corrected visual acuity

**Figure 2:**
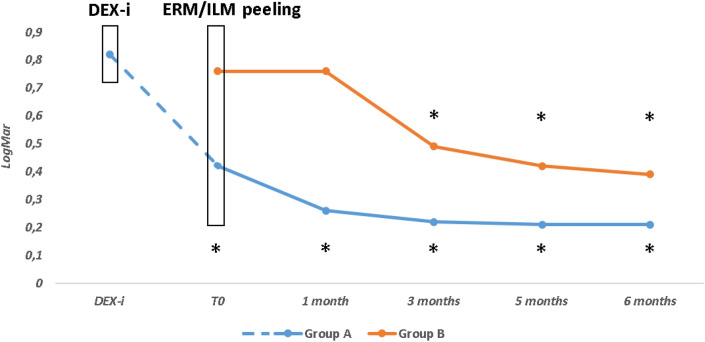
Mean Best corrected visual acuity (BCVA) in Groups (**A** and **B**) over the study follow-up. DEX-i, intravitreal dexamethasone implant; ERM/ILM, Epiretinal Membrane/Inner Limiting Membrane; *P < 0.01 as compared to T_0_ within each group.

In Group A, mean BCVA increased significantly from 20/132 (0,82 ± 0,4 LogMar) to 20/53 (0,42 ± 0,19 logMar) at 1 month after DEX-i (T_0_) (*P* < 0.01). At T_0_, visual acuity was significantly better in Group A than in B (*P* < 0.0001). In Group A, mean BCVA significantly improved at all time points (*P* < 0.001) after surgery. In Group B, mean BCVA significantly improved only from the third month and in the following months (*P* < 0.001) after surgery (Fig. 2; Table 2).

**Table 2:** Linear mixed-effects model to examine the effect of different treatments on BCVA, CRT, and IOP at different time-points (*n* = *21*).

Parameters*	Time					*p *^*¥*^			
	T_0_ *(a)*	1 month *(b)*	3 months *(c)*	5 months *(d)*	6 months *(e)*	*b vs (a)*	*c vs (a)*	*d vs (a)*	*e vs (a)*
BCVA									
*Group A*	0.42 ± 0.19	0.25 ± 0.17	0.21 ± 0.17	0.21 ± 0.17	0.21 ± 0.17	**0.002**	** < 0.001**	** < 0.001**	** < 0.001**
*Group B*	0.76 ± 0.21	0.76 ± 0.23	0.49 ± 0.16	0.42 ± 0.18	0.39 ± 0.23	0.97	** < 0.001**	** < 0.001**	** < 0.001**
*p *^*^*^	** < 0.0001**	** < 0.0001**	**0.001**	**0.01**	**0.03**				
*Mixed *^*§*^	*Treatment* ** < 0.0001**	*Time* ** < 0.0001**	*Interaction* **0.0001**						
CRT									
*Group A*	326.2 ± 63.5	277.0 ± 54.1	262.0 ± 74.1	264.0 ± 76.3	265.9 ± 84.1	** < 0.001**	** < 0.001**	** < 0.001**	** < 0.001**
*Group B*	408.5 ± 55.4	385.8 ± 57.1	309.0 ± 52.1	295.3 ± 53.0	303.4 ± 69.7	0.12	** < 0.001**	** < 0.001**	** < 0.001**
*p *^*^*^	** < 0.001**	** < 0.001**	** < 0.001**	** < 0.001**	** < 0.001**				
*Mixed *^*§*^	*Treatment* **0.02**	*Time* ** < 0.0001**	*Interaction* **0.0002**						
IOP									
*Group A*	16.09 ± 1.14	15.91 ± 1.30	16.27 ± 1.10	15.73 ± 1.35	16.91 ± 1.30	0.71	0.71	0.46	0.10
*Group B*	15.60 ± 1.71	16.10 ± 0.87	16.10 ± 0.57	15.90 ± 0.74	16.60 ± 1.07	0.33	0.33	0.56	0.05
*p *^*^*^	0.33	0.71	0.73	0.73	0.54				
*Mixed *^*§*^	*Treatment* 0.60	*Time*0.05	*Interaction* 0.84						

*As meand and standard deviation (M ± SD).

*BCVA* Best corrected visual acuity, *CRT* Central retinal thickness, *IOP* Intraocular pressure.

^^^Treatment effect for each time, ^§^ Mixed-effects, ^¥^ Contrasts of marginal linear predictions.

Significant values are in bold

The comparison among the groups revealed that the mean BCVA in Group A was significantly lower than Group B at each time point (at 1 month, *P* < 0.0001; 3 months, *P* = 0.001; 5 months, *P* = 0.01; 6 months, *P* = 0.03) (Table 2).

### Central retinal thickness

**Figure 3:**
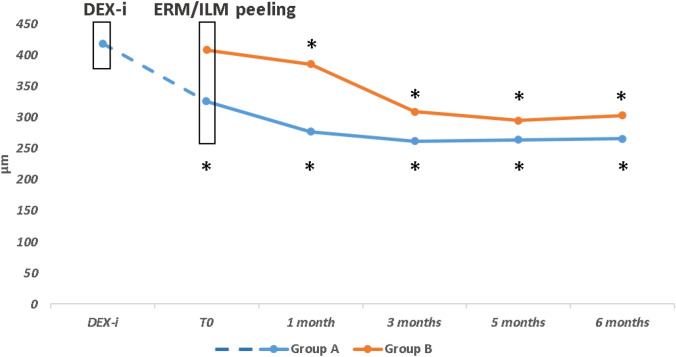
Mean Central retinal thickness (CRT) in Groups (**A** and **B**) over the study follow-up. DEX-i, intravitreal dexamethasone implant; ERM/ILM, Epiretinal Membrane/Inner Limiting Membrane; *P < 0.001 as compared to T_0_ within each group.

In Group A, mean CRT decreased from 419,1 ± 81,5 µm to 326,3 ± 63,6 µm at 1 month after DEX-i (T_0_) (*P* < 0.001). Then, a progressive thinning to 265,9 ± 84,1 µm at Month 6 after surgery (*P* < 0.001) was observed. In Group B, mean CRT decreased from 408,5 ± 55,4 µm at T_0_ to 303,4 ± 69,7 µm at Month 6 after surgery (*P* < 0.001) (Fig. 3; Table 2).

The comparison among the groups revealed that the mean CRT in Group A was significantly lower than Group B (*P* < 0.001) at each time point (Table 2).

In Group A, only 1 patient had CRT > 300 µm at month 3, showing a progressive increase of thickness over follow-up. This patient refused rescue therapy over 6 months postoperatively.

In Group B, 5 patients with CRT > 300 µm at month 3 showed a further reduction in thickness at 5 months, and only 3 of these had a new increase of thickness at 6 months. Furthermore, another 2 patients had a recurrence of edema (CRT > 300 µm) at 6 months after surgery. At the last follow-up, a second DEX-i was scheduled for 1 patient in Group A and 5 patients in Group B (*P* = 0.06).

### Intraocular pressure and complications

**Figure 4:**
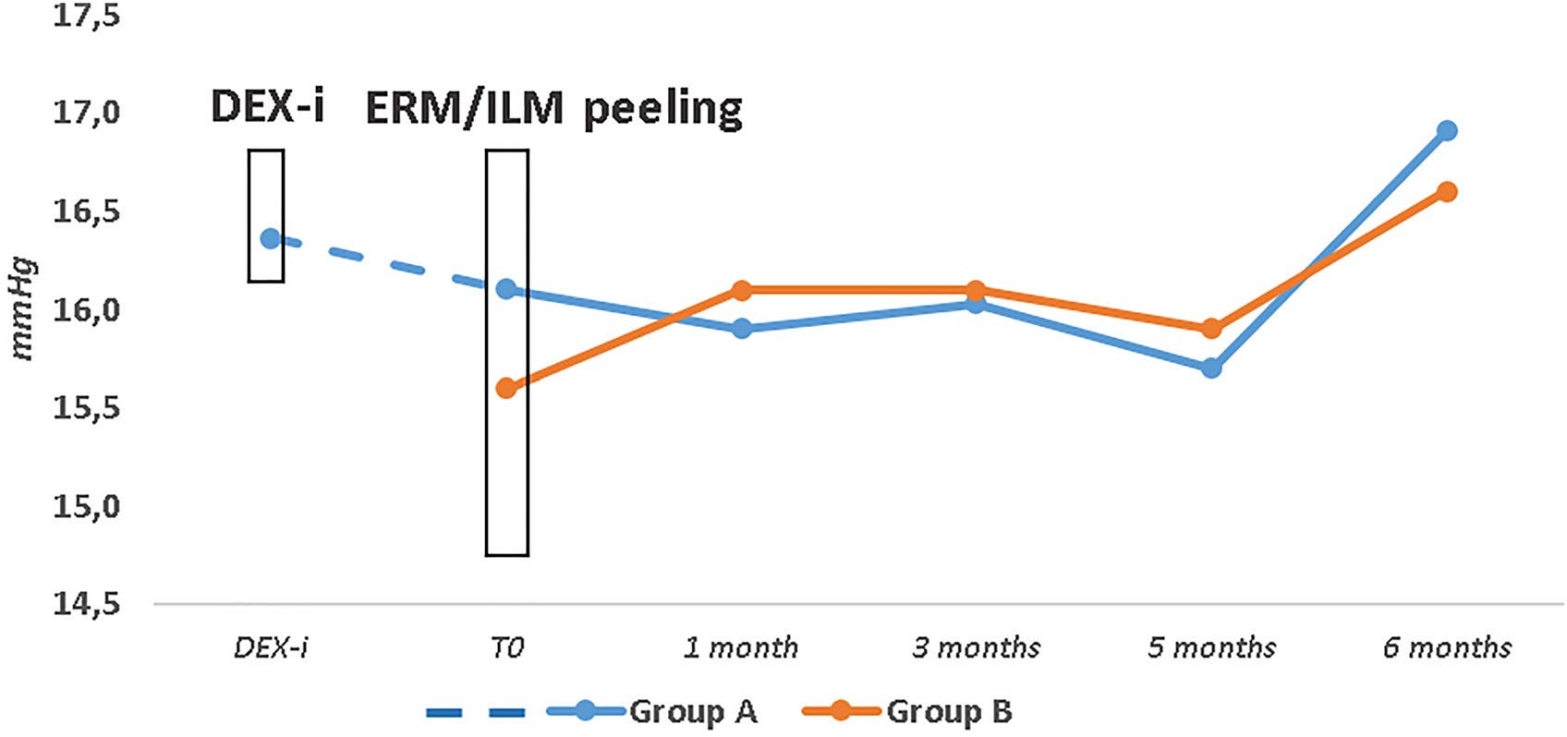
Mean Intraocular pressure (IOP) in Groups (**A** and **B**) over the study follow-up. DEX-i, intravitreal dexamethasone implant; ERM/ILM, Epiretinal Membrane/Inner Limiting Membrane.

During the study, mean IOP did not significantly increase in both groups, neither significant differences were observed between the groups (Fig. 4). None of the patients showed significant increase in the IOP requiring medical or surgical management.

Comparing the variations of the two groups, no significant differences were found over the follow-up. No surgical complications were detected.

### Linear mixed-effects model

The model revealed that the effect of each treatment, time, and interaction between treatment and time on BCVA and CRT was significantly different among the groups. The same was not the case for IOP (Table 2).

## Discussion

This study analyzed the efficacy and safety of macular peeling preceded by DEX-i one month before surgery to treat tDME.

Clinical characteristics such as a long duration of diabetes and high HbA1c level at the time of surgery, preoperative presence of ERM, and poor baseline visual acuity have been associated with poor prognosis in patients with DME undergoing vitreoretinal surgery^33^. So our functional results should be analyzed considering that the mean duration of diabetes mellitus was not different between the groups, ranging from 16 to 18 years, the mean HbA1c level before surgery was 7% in both groups, and the mean visual acuity before any treatment was not significantly different between the groups.

The efficacy and safety of DEX-i for various retinal diseases have been proved in clinical trials and real-life studies^34–36^. Steroids have multiple mechanisms of action, inhibiting different molecules involved in vascular permeability and inflammation processes^37^. Several authors reported significant anatomical and functional effects of DEX-i in vitrectomized eyes with different conditions but implanted at the time of vitrectomy or after vitrectomy^38–41^. To the best of our knowledge, there are no published papers evaluating the impact of ERM and ILM peeling performed during the time-release of DEX-i on eyes affected by tDME. Comparing the changes in outcomes due to the combined approach versus peeling alone, we speculated on the role of DEX-i in inhibiting the inflammatory molecules related to DME and containing the inflammatory stress due to the surgical maneuvers, obtaining better and earlier results than peeling alone, and extending them over time.

In implanted eyes, preoperative CRT ranged from 311 to 610 µm, suggesting the heterogeneity of DME feature as a target for DEX implant, also in tDME. The greatest mean reduction in CRT occurred just after one month from the implant when dexamethasone reaches the highest concentration in the vitreous humor, only partially removed for macular peeling, followed by a stabilization of retinal thickness during the next months, and a late macular thickening (3–35 µm), in line with the known pharmacodynamics of Ozurdex®^42^. From T_0_ to 6 months, a significant reduction in CRT was observed in both groups. However, the implanted eyes arrived at the surgery with a thinner central retina than non-implanted eyes and maintained a thinner macula (mean value, < 300 µm) overall follow-up when compared to the non-implanted eyes that reached the lowest mean CRT (295 µm) only at 5 months. Overall, DEX-i combined with ERM/ILM peeling gave a mean reduction of 153 µm vs. 105 µm after peeling alone. Implanted eyes had a progressive and regular reduction of retinal thickness after surgery, while non-implanted eyes achieved a significant reduction only after 3 months, with the greatest change between the first and the third month. The vitreous body, together with the posterior hyaloid and the ILM, may be involved in the pathogenesis of DME^43,44^. Chronic inflammation due to hypoxia, oxidative stress, and upregulation of VEGF^45,46^ induces the adhesion of the posterior vitreous cortex to the ILM and promotes the proliferation of Müller cells, myofibroblast-like cells, and macrophages as the main components of ERM associated with DME^45–47^. ERM and ILM peeling may act on macular edema by removing tractional forces on the retina^48^, improving oxygen diffusion through the vitreous^49^, and removing the largest reservoir of pro-inflammatory factors^50^. This way of acting of macular peeling justifies the significant reduction of CRT observed in both groups, as previously reported by several studies on ILM peeling for tDME^16,51,52^. Furthermore, DEX-i releases active ingredients within the vitreous chamber over 3–6 months, potentially limiting the inflammatory stress of surgical maneuvers on retinal tissue and quickening anatomical recovery.

In Group A, the greater mean visual improvement (0.4 LogMAR) occurred just after one month from the implant, followed by further functional recovery (0.21 LogMAR) up to the sixth month after surgery. In Group B, the mean visual acuity increased only at month 3 after surgery and then remained substantially stable with an overall average gain of 0.37 LogMAR.

The anatomical outcome is better than functional results in eyes with tDME that underwent surgery^15–17^. On a large cohort, between 28 and 49% of eyes had an improvement in visual acuity, whereas between 13 and 31% had a worsening in function after surgery^17^. However, we observed a visual acuity improvement in 10/11 of implanted eyes and 8/10 of non-implanted eyes at the last follow-up. Only in one eye that underwent peeling alone a worsening in visual acuity was observed.

Probably, the better predictive factors for visual recovery observed in our cohort than that observed in the above-mentioned cohort including the preoperative retinal thickness (median, 364 µm vs. 491µm^17^) and the preoperative visual acuity (median, 20/66 vs. 20/100^17^) could explain our better functional results.

In the linear mixed-effects model, we have evaluated the effect of each treatment, the effect of time, and the effect of the interaction between treatment and time. For repeated measurements of BCVA and CRT, significant differences among the treatments were observed for distinct time points. Before and after surgery, the implanted eyes always had better outcomes than non-implanted eyes. At the time of surgery, some predictive factors influencing the efficacy of macular peeling, including preoperative visual acuity and retinal thickness, were significantly better in eyes previously implanted.

ILM peeling appeared to extend the benefit of the DEX-i in eyes with DME^53^ and tDME^28^ and reduce the rate of reimplanting^28^. Furthermore, a recent meta-analysis proved that ILM peeling combined with the removal of ERM could reduce the recurrence rate of ERM^54^ by eliminating a scaffold for proliferating cells, which could stimulate a leukocyte response in the macular region causing persistent macular edema^55^. The recurrence of macular edema requiring a second DEX-i was observed for only one patient previously implanted. In contrast, five out of ten patients not previously implanted had a final CRT > 300 µm, so potentially needing rescue therapy with DEX-i.

As known, ILM peeling may cause damage to the Müller cells and it could explain the cases with the increase of macular thickness^56^, potentially worsened by diabetic retinopathy.

Regarding safety concerns, none of the patients in the DEX-i group showed a significant increase in the IOP requiring medical or surgical management. Additionally, no statistically significant differences in the change of IOP were recorded between the groups throughout the follow-up. Furthermore, the study selection criteria allowed us to select patients with a clear vitreous, so in all implanted eyes, DEX-i was always clearly visible in the vitreous chamber, not hindering the vitrectomy maneuvers.

This study has some limitations that should be noted; among them is its retrospective design. Other limitations are the single-center nature of the study, the limited number of patients, and the absence of analysis of retinal layers integrity and its relationship with visual function. Moreover, another limitation of the study is the absence of adjustments for multiplicity, and as such, all analyses should be regarded as exploratory.

In conclusion, our results seemed to confirm the efficacy of ERM/ILM peeling to treat tDME. DEX-i performed one month before surgery seemed to be a safe approach and ensure a greater and faster recovery considering functional and tomographic parameters.

## Data Availability

The datasets generated during and/or analysed during the current study are available from the corresponding author on reasonable request.
